# The impact of glycated hemoglobin trajectories on hypertension risk: a retrospective cohort study

**DOI:** 10.3389/fnut.2025.1680891

**Published:** 2025-11-05

**Authors:** Yongbing Sun, Yu Shen, Ao Liu, Caiwen Zhao, Xiaoqi Ji, Xin Li, Zhi Zou, Zhonglin Li, Xiaoling Wu, Yong Wang, Min Liu, Yongli Li, Yibin Hao

**Affiliations:** ^1^Department of Medical Imaging, Zhengzhou University People's Hospital, Henan Provincial People's Hospital, Zhengzhou, Henan, China; ^2^The Third Affiliated Hospital of Zhengzhou University, Zhengzhou, Henan, China; ^3^Department of Medical Imaging, Henan University People's Hospital, Henan Provincial People's Hospital, Zhengzhou, China; ^4^Department of Nuclear Medicine, Henan Provincial People's Hospital, Zhengzhou, Henan, China; ^5^Henan Provincial People's Hospital, Zhengzhou, Henan, China; ^6^Department of Hypertension, Henan Provincial People's Hospital, Zhengzhou, Henan, China; ^7^Chronic Health Management Laboratory, Department of Health Management, Henan Provincial People’s Hospital, Zhengzhou, Henan, China

**Keywords:** glycated hemoglobin, hypertension, trajectory analysis, prospective longitudinal study, health screening

## Abstract

**Background:**

Glycated hemoglobin (HbA1c) reliably reflects long-term glucose control and has been linked to hypertension development. This study investigates the relationship between baseline HbA1c levels, HbA1c trajectories, and hypertension risk.

**Methods:**

This retrospective cohort study included 10,138 adults from health screenings at Henan Provincial People’s Hospital (January 2018–January 2025). Mean age was 54.03 ± 12.97 years, with 31.44% women and mean follow-up of 43.92 months. We analyzed hypertension incidence across HbA1c groups using Kaplan–Meier curves and identified HbA1c trajectory patterns using latent class trajectory modeling (LCTM). Cox proportional hazards models evaluated associations between baseline HbA1c tertiles, HbA1c trajectories, and hypertension risk. Restricted cubic splines explored dose–response relationships.

**Results:**

During follow-up, 3,452 participants (34.05%) developed hypertension. After adjustment, participants in the highest baseline HbA1c tertile had significantly increased hypertension risk versus the lowest tertile (HR = 1.49, 95%CI: 1.31–1.70). LCTM identified three distinct trajectories: low-stable (5.57 ± 0.36%), medium-stable (6.45 ± 0.59%), and high-stable (8.42 ± 1.39%). Compared to low-stable trajectory, medium-stable and high-stable groups showed significantly increased risks (HR = 1.38, 95%CI: 1.24–1.53; HR = 2.71, 95%CI: 2.21–3.32, respectively). Restricted cubic spline analysis revealed a J-shaped relationship with an inflection point at HbA1c = 5.70% (*P* for nonlinearity < 0.001).

**Conclusion:**

Elevated baseline HbA1c levels, particularly above 5.70%, and medium-to-high stable HbA1c trajectories significantly increase hypertension risk among adults undergoing health screening. HbA1c could serve as a valuable biomarker for hypertension risk assessment.

## Introduction

Hypertension is a leading risk factor for cardiovascular diseases, posing a significant global public health challenge (1). The global prevalence has reached approximately 33%, affecting 1.3 billion individuals worldwide and causing more than 10 million deaths annually (2). In China, approximately 245 million adults are affected, highlighting the urgent need for effective risk stratification strategies (3). The development of hypertension involves complex interactions among multiple metabolic disturbances, with glucose metabolism playing a crucial role (4). Specifically, high blood sugar levels lead to oxidative stress, reducing the availability of nitric oxide, which impairs blood vessel function (5). Additionally, elevated glucose promotes the formation of advanced glycation end products (AGEs), causing blood vessels to stiffen (6). Insulin resistance further raises blood pressure by activating both the sympathetic nervous system and the renin-angiotensin-aldosterone system (RAAS) (7). Thus, monitoring blood glucose is not only essential for diabetes prevention but may also have significant clinical value in assessing hypertension risk.

Glycated hemoglobin (HbA1c), one of the most important types of hemoglobin, reliably reflects average blood glucose levels over the previous 8–12 weeks (8). Compared with fasting blood glucose (FBG), HbA1c provides a more comprehensive picture of long-term glucose control, is less affected by temporary fluctuations, and is thus considered stable and reliable (9). It serves as a primary indicator for assessing the quality of diabetes management in healthcare settings (10). Diabetes commonly coexists with hypertension, and previous studies have established a strong relationship between elevated HbA1c levels and increased risks of both diabetes and hypertension (11, 12). A cross-sectional study involving 1,462 non-diabetic Chinese individuals found that higher HbA1c levels significantly increased the risk of hypertension and isolated systolic hypertension (13). Prospective studies have also confirmed this strong relationship. For instance, a longitudinal study using data from the China Health and Nutrition Survey (*n* = 4,074) identified a significant association between HbA1c and hypertension risk (14). Similarly, recent data from the U. S. National Health and Nutrition Examination Survey (NHANES, 2011–2018; *n* = 10,503) demonstrated an independent association between HbA1c and adult hypertension risk (15). Nevertheless, most studies have only measured HbA1c at a single time point, ignoring the potential significance of changes in HbA1c over time (16). Additionally, previous research typically examined baseline HbA1c or trajectories separately, limiting a comprehensive understanding of how HbA1c influences hypertension risk. An integrated approach—combining immediate risk assessment based on baseline HbA1c and long-term evaluation through trajectory patterns—can offer deeper insights into the role of HbA1c in the development and progression of hypertension.

To address this research gap, our team utilized a 7-year longitudinal dataset from the Health Screening Center of Henan Provincial People’s Hospital. Using advanced latent class trajectory modeling (LCTM), we systematically assessed the association between baseline HbA1c levels, their dynamic trajectories, and the risk of developing hypertension. This study aims not only to clarify the potential value of HbA1c as a biomarker for predicting hypertension risk but also to explore its practical implications for clinical risk stratification and early preventive interventions. Ultimately, the results could provide theoretical and empirical foundations for developing innovative cardiovascular prevention strategies based on dynamic monitoring of metabolic indicators.

## Materials and methods

### Study design and population

**Figure 1:**
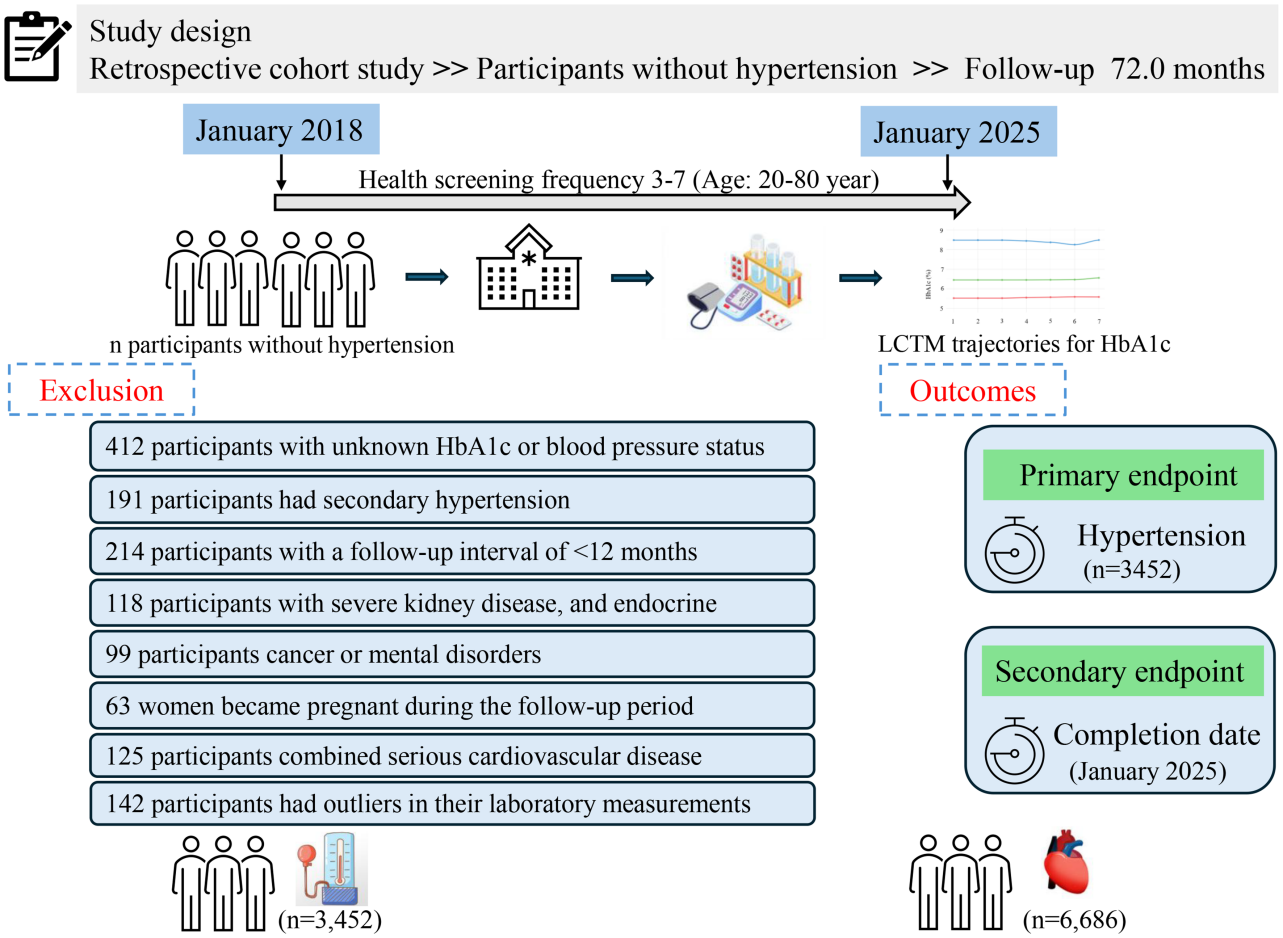
Study design and participant flow diagram.

This retrospective cohort study utilized real-world data collected from adults aged 20–80 years who underwent routine health screenings at the Health Management Center of Henan Provincial People’s Hospital between January 2018 and January 2025. All participants were registered members (employees or their parents) benefiting from employer-sponsored annual health check-ups, thus maintaining a stable long-term relationship with the center. Medical examinations occurred every 6 to 12 months, and each participant had a minimum of three and up to seven recorded visits. Follow-up commenced from the initial health examination. Participants diagnosed with hypertension during the study period ceased follow-up upon diagnosis, while those who remained free of hypertension continued follow-up until January 31, 2025. During the initial screening, 412 individuals were excluded due to missing or insufficient data regarding HbA1c or blood pressure status. Additionally, another 952 participants were excluded for the following reasons: secondary hypertension (n = 201), a follow-up duration of less than 12 months (n = 217), occurrence of severe liver or kidney disease during follow-up (defined as liver enzyme levels greater than three times the upper limit or an eGFR less than 60 mL/min/1.73 m^2^), endocrine disorders potentially influencing glucose metabolism (*n* = 138), diagnosis of cancer or psychiatric conditions during follow-up (*n* = 99), pregnancy during follow-up (*n* = 132), or severe cardiovascular diseases requiring hospitalization (*n* = 165). Consequently, the final analysis included 10,138 participants who were free from hypertension at baseline and had complete medical records throughout the follow-up period. A detailed description of the study design and participant flow is illustrated in Figure 1.

### Laboratory measurements

All investigators underwent standardized training to ensure objectivity and accuracy throughout the study. A unique identification number was assigned to each participant, allowing precise matching across health examination records, medical histories, and questionnaire responses. Data collection strictly followed predefined standard operating procedures. Before each medical examination, demographic and health-related information—including smoking status, alcohol consumption, history of endocrine, liver, or kidney disease, cancer, and the use of medications for diabetes or lipid disorders—was collected via a structured questionnaire. Following completion, two trained validation personnel independently reviewed, compiled, and verified the collected data. The database was regularly maintained and subjected to quality audits. Missing or erroneous data were resolved by directly contacting participants either face-to-face or via telephone verification.

All participants provided fasting venous blood samples at 8:00 a.m. after a minimum of 12 h overnight fasting. Most biochemical parameters—including blood urea nitrogen (BUN), creatinine (Cre), FBG, total cholesterol (TC), low-density lipoprotein cholesterol (LDL-C), triglycerides (TG), and high-density lipoprotein cholesterol (HDL-C)—were measured using an Olympus AU 5400 automatic biochemical analyzer (Olympus, Shizuoka, Japan). Blood cell parameters such as lymphocytes, neutrophils, and white blood cells (WBC) were measured using a Beckman Coulter DxH800 automatic hematology analyzer (Beckman Coulter, Brea, United States). All laboratory analyses were conducted according to standardized laboratory protocols.

### Assessment of HbA1c

HbA1c levels were measured by high-performance liquid chromatography using the Lifotronic H9 HbA1c analyzer (Lifotronic, Shanghai, China). Two complementary analytical approaches were applied in this study: baseline HbA1c tertiles were used for immediate risk stratification, and trajectory analysis was conducted to assess long-term changes in risk. Baseline HbA1c was categorized into tertiles to evaluate initial risk distribution, while trajectory modeling captured dynamic HbA1c changes over the follow-up period. Baseline HbA1c values were derived from each participant’s initial blood glucose assessment. The mean HbA1c for each participant was calculated as the arithmetic average of individual HbA1c measurements obtained during follow-up visits from January 2018 to January 2025. LCTM was used to categorize participants into distinct HbA1c trajectory groups based on longitudinal patterns.

### Assessment of blood pressure

Blood pressure measurements were conducted by endocrinologists at the Health Management Center. Participants fasted for at least 12 h prior to measurement. Following a resting period of 5 min, systolic blood pressure (SBP) and diastolic blood pressure (DBP) were measured twice using an automated electronic blood pressure monitor (Omron U30, Kyoto, Japan) with the participant’s right arm positioned at heart level in a semi-flexed state. The mean value of these two measurements was used for analysis.

### Outcome

The primary outcome of this study was the development of hypertension, with the study endpoint defined as the date of hypertension diagnosis. Participants without hypertension were followed until the study’s secondary endpoint, which was January 31, 2025. Hypertension was diagnosed according to established Chinese guidelines for adults (17). Diagnosis was confirmed if any of the following criteria were met: (1) two consecutive blood pressure measurements showing systolic blood pressure (SBP) ≥ 140 mmHg or diastolic blood pressure (DBP) ≥ 90 mmHg; (2) current use of antihypertensive medications, initially obtained through self-report and subsequently verified through our hospital’s electronic medical record system; or (3) undergoing treatment specifically for hypertension. If hypertension was not immediately evident, confirmation required two abnormal measurements taken on separate days within a one-week interval. For participants with only one abnormal result, a second confirmatory blood pressure measurement was taken within 1–2 weeks. Any inconsistent reading prompted repeated measurements for verification. Participants who did not meet these criteria were classified as non-hypertensive. All diagnoses were verified by cross-checking medical records within the hospital’s electronic health record system.

### Definitions of variables

BMI was = weight (kg)/height^2^ (m^2^).

Current smoking was defined as self-reported consumption of at least one cigarette per day maintained throughout the previous 12 months. Current drinking was defined as the consumption of alcoholic beverages at a minimum frequency of once per week during the 12-month period preceding health examination.

Estimated glomerular filtration rate (eGFR) was calculated using the formula: eGFR (mL/min/1.73 m^2^) = 175 * serum creatinine^−1.154^ * age^-0.203^ * 0.742 (if female) * 1.212 (if black) (18).

### Handling of missing data

In this study, the overall proportion of missing data was low. Completeness analysis revealed that missing rates for all key covariates were below 5%, with marriage status having the highest missing rate (4.2%), followed by BMI (3.8%). We employed multiple imputation using Markov Chain Monte Carlo (MCMC) methods to address missing data, generating 10 complete datasets. The imputation model incorporated all study covariates, primary exposure variables, outcome variables, and their interactions to ensure accuracy of the imputation process. Rubin’s rules were applied to pool results from the multiple imputations for the final analysis. To assess the potential impact of missing data on our findings, we conducted sensitivity analyses comparing results from complete case analysis with those from multiple imputations. These analyses demonstrated high consistency in point estimates and 95% confidence intervals between the two approaches (differences <5%), indicating the robustness of our results to the missing data handling method. For the HbA1c trajectory analysis, we established inclusion criteria requiring participants to have at least 3 valid HbA1c measurements with intervals ≥6 months during the follow-up period to ensure reliable trajectory identification. Ultimately, 97.8% of participants met these criteria and were included in the trajectory analysis.

### Statistical analysis

All statistical analyses in this study were performed using R software (version 4.4.1, R Foundation). All statistical tests were two-sided, with a significance threshold of *p* < 0.05. The primary R packages used were: ‘lcmm’ for latent class trajectory modeling, ‘survival’ for Kaplan–Meier survival analysis and Cox proportional hazards models, ‘survive’ for visualization of survival curves, ‘rms’ and ‘splines’ for constructing restricted cubic spline models, and ‘ggplot2’ for data visualization and figure preparation.

Normality of all continuous variables was assessed. Variables conforming to a normal distribution were expressed as means ± standard deviations, while non-normally distributed variables were summarized as medians (interquartile ranges). Differences between groups were evaluated using either *t*-tests or rank-sum tests, as appropriate. Categorical variables were expressed as counts and percentages, with between-group comparisons conducted using chi-square tests.

Baseline HbA1c was categorized into tertiles (T1, T2, and T3), with T1 representing the lowest values and T3 the highest. The follow-up duration spanned from the baseline assessment to the final recorded visit, encompassing biochemical data and hypertension diagnoses. LCTM was applied to evaluate longitudinal HbA1c patterns over the follow-up period (19). We tested models with 2–5 trajectory groups with linear, quadratic, and cubic terms to identify the optimal number of trajectory groups. The final model selection was based on multiple criteria: (1) the Bayesian Information Criterion, with lower values indicating better fit; (2) mean posterior probability of trajectory group membership ≥0.7 for all groups; (3) reasonable group sizes (each group containing at least 5% of the total sample); (4) distinct trajectory patterns with minimal overlap of 95% confidence intervals; and (5) clinical interpretability. Additionally, model adequacy was evaluated by assessing classification accuracy using odds of correct classification (OCC > 5 indicating good assignment accuracy) and comparing model-estimated group proportions to observed group proportions. Model parameters were estimated using the expectation–maximization algorithm with maximum likelihood estimation. Models with 2–5 trajectory groups were systematically compared using established statistical criteria. The three-group model was ultimately selected based on optimal statistical performance (BIC = −186,891.5, entropy = 0.863, mean posterior probability = 0.82) and superior clinical interpretability, with all groups maintaining adequate sample sizes and distinct trajectory patterns.

To facilitate comparison and validate the primary findings, Kaplan–Meier survival curves were constructed separately for baseline HbA1c tertile groups and trajectory groups, with differences assessed using log-rank tests. Multivariable Cox proportional hazards models were utilized to investigate associations between HbA1c and hypertension risk. Three distinct models were developed: an unadjusted model; Model I, adjusted for demographic factors (sex, age, ethnicity, and marital status); and Model II, comprehensively adjusted for all identified potential confounders. Confounding variables were selected by excluding factors with variance inflation factors (VIF) greater than 10. Restricted cubic spline analysis was performed within Cox models to explore dose–response relationships between baseline HbA1c, HbA1c trajectories, and hypertension risk. To ensure the robustness of our findings, three sensitivity analyses were performed: (1) inclusion of mean HbA1c as a predictor in the fully adjusted Cox model, (2) repeating the Cox analyses after excluding participants taking antihyperlipidemic and lipid-lowering medications, and (3) conducting sex-stratified subgroup analyses and testing for interaction between HbA1c and sex, given the relatively lower proportion of female participants (31.44%) in our cohort, and (4) logistic regression analyses to address potential time bias in hypertension onset detection inherent in health screening-based studies, using hypertension occurrence as a binary outcome variable with the same covariate adjustments as the Cox models. Furthermore, the predictive performance of baseline HbA1c tertiles versus HbA1c trajectory groups was compared based on incidence rates and hazard ratios for hypertension.

### Ethics declarations

Adhering to the principles outlined in the Declaration of Helsinki, our retrospective longitudinal analysis was conducted following approval from the Henan Provincial People’s Hospital Ethics Committee (Approval Number: 2021 Ethical Review No. 68). Due to the retrospective nature of the study, the ethics committee waived the requirement for informed consent. The study protocol included implementing re-coding procedures for identifying information to safeguard anonymity of personal data.

## Results

### Baseline characteristics by baseline HbA1c tertiles

To comprehensively evaluate the relationship between HbA1c and hypertension risk, we first examined the association between baseline HbA1c levels and hypertension incidence. The study included 10,138 adults who underwent routine health screenings, with an average age of 54.03 ± 12.97 years; among them, 3,187 (31.44%) were women. The overall mean baseline HbA1c level for the cohort was 5.91 ± 0.83%. Detailed baseline characteristics of all participants are summarized in Supplementary Table S1. Participants were categorized into tertiles (T1, T2, and T3) based on baseline HbA1c levels. The T1 and T2 groups consisted predominantly of individuals with normal to mildly elevated glucose levels, with 31.8% of T2 participants approaching prediabetic status (HbA1c ≥ 5.70%), and both groups having blood pressure levels ranging from optimal to prehypertensive. Compared to the T1 and T2 groups, participants in the highest HbA1c tertile (T3) were significantly older, predominantly male, had higher BMI, were more frequently married, and reported greater use of antihyperlipidemic and lipid-lowering medications (all *p* < 0.001). Furthermore, the T3 group exhibited significantly higher levels of BUN, Cre, FBG, lymphocytes, neutrophils, WBC, TC, LDL-C, and TG, alongside lower eGFR and HDL-C, compared to the other groups (all *p* < 0.001). Additionally, the incidence of hypertension in the T3 group (48.85%) was significantly higher than that observed in the T2 (28.42%) and T1 (21.84%) groups. These findings are comprehensively summarized in Table 1.

**Table 1:** Baseline characteristics by the baseline HbA1c tertiles.

Characteristics	T1 (3.70–5.40)	T2 (5.50–5.90)	T3 (6.00–13.30)	*p*-value
*N*	2,628	3,870	3,640	
HbA1c	5.19 ± 0.22	5.69 ± 0.14	6.73 ± 1.01	<0.001
Mean HbA1c	5.30 ± 0.29	5.66 ± 0.26	6.60 ± 0.98	<0.001
Age, years	47.99 ± 11.94	52.78 ± 12.09	59.73 ± 12.24	<0.001
Sex, *n* (%)				<0.001
Female	936 (35.62)	1,264 (32.66)	987 (27.12)	
Male	1,692 (64.38)	2,606 (67.34)	2,653 (72.88)	
Ethnic group, *n* (%)				0.039
Non-Han	31 (1.18)	60 (1.55)	33 (0.91)	
Han	2,597 (98.82)	3,810 (98.45)	3,607 (99.09)	
Marriage status, *n* (%)				<0.001
Unmarried	87 (3.31)	50 (1.29)	17 (0.47)	
Married	2,541 (96.69)	3,820 (98.71)	3,623 (99.53)	
Current drinking, *n* (%)				0.684
No	2,067 (78.65)	3,024 (78.14)	2,874 (78.96)	
Yes	561 (21.35)	846 (21.86)	766 (21.04)	
Current smoking, *n* (%)				0.513
No	2,180 (82.95)	3,252 (84.03)	3,040 (83.52)	
Yes	448 (17.05)	618 (15.97)	600 (16.48)	
Antihyperlipidemic agents, *n* (%)				<0.001
No	2,617 (99.58)	3,827 (98.89)	3,543 (97.33)	
Yes	11 (0.42)	43 (1.11)	97 (2.67)	
Lipid-lowering medications, *n* (%)				<0.001
No	2,601 (98.97)	3,809 (98.42)	3,510 (96.43)	
Yes	27 (1.03)	61 (1.58)	130 (3.57)	
BMI, kg/m^2^	24.03 ± 2.95	24.51 ± 2.95	25.25 ± 2.98	<0.001
SBP, mmHg	119.99 ± 14.09	122.82 ± 15.16	130.45 ± 18.03	<0.001
DBP, mmHg	72.83 ± 10.14	73.87 ± 10.23	75.67 ± 10.75	<0.001
BUN, mmol/L	4.93 ± 1.20	5.14 ± 1.29	5.54 ± 1.55	<0.001
Cre, μmol/L	65.75 ± 18.69	66.20 ± 15.39	67.13 ± 22.38	0.012
eGFR, mL/min/1.73m^2^	112.74 ± 20.40	110.38 ± 20.30	109.52 ± 23.54	<0.001
FBG, mmol/L	4.64 ± 0.48	4.81 ± 0.50	6.01 ± 1.77	<0.001
Lymphocyte, 10^9^/L	1.80 ± 0.50	1.89 ± 0.66	2.01 ± 1.48	<0.001
Neutrophil, 10^9^/L	3.14 ± 0.98	3.22 ± 1.02	3.48 ± 1.09	<0.001
WBC, 10^9^/L	5.42 ± 1.31	5.63 ± 1.43	6.07 ± 2.07	<0.001
TC, mmol/L	4.78 ± 0.84	4.90 ± 0.93	4.80 ± 1.05	<0.001
LDL-C, mmol/L	2.75 ± 0.68	2.88 ± 0.77	2.83 ± 0.86	<0.001
TG, mmol/L	1.29 (0.96–1.82)	1.42 (1.05–1.98)	1.60 (1.17–2.26)	<0.001
HDL-C, mmol/L	1.37 ± 0.30	1.33 ± 0.29	1.25 ± 0.28	<0.001
Time, month	44.72 ± 14.76	43.99 ± 13.83	43.27 ± 12.86	<0.001
Hypertension, *n* (%)				<0.001
No	2,054 (78.16)	2,770 (71.58)	1,862 (51.15)	
Yes	574 (21.84)	1,100 (28.42)	1,778 (48.85)	

HbA1c, glycosylated hemoglobin; BMI, body mass index; SBP, systolic blood pressure; DBP, diastolic blood pressure; BUN, blood urea nitrogen; Cre, Creatinine; eGFR, estimated glomerular filtration rate; FBG, fasting blood glucose; WBC, white blood cell; TC, total cholesterol; LDL-C, low-density lipoprotein cholesterol; TG, triglycerides; HDL-C, high-density lipoprotein cholesterol. Except for TG is expressed as medians (upper and lower quartiles), all other variables are expressed as mean ± standard deviation or counts (percentages).

### Baseline characteristics stratified by HbA1c trajectory groups

**Figure 2:**
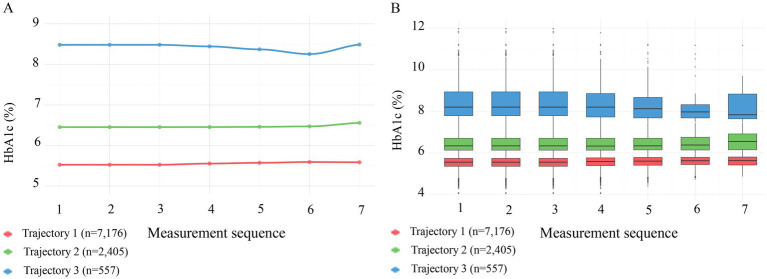
Patterns of HbA1c across three trajectory groups during follow-up. **(A)** Line graphs showing mean HbA1c levels across 7 sequential measurements. Trajectory 1 (red, *n* = 7,176) maintained a low level of ~5.5%; Trajectory 2 (green, *n* = 2,405) maintained a medium level of ~6.5%; and Trajectory 3 (blue, *n* = 557) maintained a high level of ~8.3%. **(B)** Box plots illustrating the distribution of HbA1c across the three trajectory groups, confirming that the groups maintained stable and distinct HbA1c distributions throughout the follow-up period.

Although baseline HbA1c levels provide immediate insight into hypertension risk, this study further investigated how longitudinal HbA1c trajectories influence hypertension development. Using LCTM, we identified three distinct HbA1c trajectory groups during the follow-up period: the low-stable group (trajectory 1, *n* = 7,176), medium-stable group (trajectory 2, *n* = 2,405), and high-stable group (trajectory 3, *n* = 557) (Figure 2). Table 2 summarizes the baseline demographic and clinical characteristics of each HbA1c trajectory group. All variables differed significantly among the trajectory groups (*p* < 0.001), except ethnicity, current drinking, and current smoking status. Specifically, compared to participants in the low-stable HbA1c group, those in the high-stable group were significantly older, had higher BMI, a higher proportion of males, and a greater percentage were married (all *p* < 0.001). Additionally, the high-stable group exhibited significantly higher levels of BUN, Cre, FBG, lymphocytes, neutrophils, WBC, TG, and significantly lower levels of TC, LDL-C, and HDL-C (all *p* < 0.001). Hypertension incidence varied substantially across trajectory groups: 26.77% in the low-stable group, 48.36% in the medium-stable group, and 66.07% in the high-stable group. Participants in the high-stable HbA1c trajectory group demonstrated the highest incidence of hypertension.

**Table 2:** Baseline characterization according to HbA1c trajectories.

Characteristics	Trajectory 1	Trajectory 2	Trajectory 3	*p*-value
*N*	7,176	2,405	557	
HbA1c	5.57 ± 0.36	6.45 ± 0.59	8.42 ± 1.39	<0.001
Mean HbA1c	5.52 ± 0.29	6.45 ± 0.39	8.48 ± 0.94	<0.001
Age, years	51.24 ± 12.38	60.41 ± 11.69	62.54 ± 12.23	<0.001
Sex, *n* (%)				<0.001
Female	2,440 (34.00)	651 (27.07)	96 (17.24)	
Male	4,736 (66.00)	1,754 (72.93)	461 (82.76)	
Ethnic group, *n* (%)				0.172
Non-Han	97 (1.35)	21 (0.87)	6 (1.08)	
Han	7,079 (98.65)	2,384 (99.13)	551 (98.92)	
Marriage status, *n* (%)				<0.001
Unmarried	145 (2.02)	9 (0.37)	0 (0.00)	
Married	7,031 (97.98)	2,396 (99.63)	557 (100.00)	
Current drinking, *n* (%)				0.419
No	5,614 (78.23%)	1,912 (79.50%)	439 (78.82%)	
Yes	1,562 (21.77%)	493 (20.50%)	118 (21.18%)	
Current smoking, *n* (%)				0.553
No	5,997 (83.57%)	2,001 (83.20%)	474 (85.10%)	
Yes	1,179 (16.43%)	404 (16.80%)	83 (14.90%)	
Antihyperlipidemic agents, *n* (%)				<0.001
No	7,126 (99.30)	2,350 (97.71)	509 (91.38)	
Yes	50 (0.70)	55 (2.29)	48 (8.62)	
Lipid-lowering medications, *n* (%)				<0.001
No	7,117 (99.18)	2,332 (96.96)	471 (84.56)	
Yes	59 (0.82)	73 (3.04)	86 (15.44)	
BMI, kg/m^2^	24.35 ± 2.96	25.38 ± 3.00	25.37 ± 2.87	<0.001
SBP, mmHg	122.52 ± 15.48	129.71 ± 17.32	133.55 ± 19.36	<0.001
DBP, mmHg	73.79 ± 10.33	75.13 ± 10.39	75.42 ± 11.82	<0.001
BUN, mmol/L	5.07 ± 1.26	5.56 ± 1.59	5.83 ± 1.61	<0.001
Cre, μmol/L	65.90 ± 16.06	66.32 ± 25.93	67.93 ± 18.24	<0.001
eGFR, mL/min/1.73m^2^	111.36 ± 20.61	107.95 ± 22.60	113.70 ± 27.51	<0.001
FBG, mmol/L	4.75 ± 0.49	5.75 ± 1.12	8.52 ± 2.55	<0.001
Lymphocyte, 10^9^/L	1.87 ± 0.93	2.00 ± 1.26	2.03 ± 0.68	<0.001
Neutrophil, 10^9^/L	3.20 ± 1.00	3.47 ± 1.09	3.73 ± 1.22	<0.001
WBC, 10^9^/L	5.58 ± 1.58	6.04 ± 1.90	6.38 ± 1.70	<0.001
TC, mmol/L	4.87 ± 0.90	4.78 ± 1.07	4.60 ± 1.10	<0.001
LDL-C, mmol/L	2.86 ± 0.74	2.80 ± 0.86	2.61 ± 0.84	<0.001
TG, mmol/L	1.38 (1.02–1.91)	1.62 (1.19–2.30)	1.68 (1.23–2.59)	<0.001
HDL-C, mmol/L	1.34 ± 0.30	1.24 ± 0.27	1.18 ± 0.28	<0.001
Time, month	43.79 ± 13.88	44.75 ± 13.52	42.07 ± 12.93	<0.001
Hypertension, *n* (%)				<0.001
No	5,255 (73.23)	1,242 (51.64)	189 (33.93)	
Yes	1,921 (26.77)	1,163 (48.36)	368 (66.07)	

Trajectory 1, Low gradual trajectory; Trajectory 2, Middle gradual trajectory; Trajectory 3, High gradual trajectory; HbA1c, glycosylated hemoglobin; BMI, body mass index; SBP, systolic blood pressure; DBP, diastolic blood pressure; BUN, blood urea nitrogen; Cre, Creatinine; eGFR, estimated glomerular filtration rate; FBG, fasting blood glucose; WBC, white blood cell; TC, total cholesterol; LDL-C, low-density lipoprotein cholesterol; TG, triglycerides; HDL-C, high-density lipoprotein cholesterol. Except for TG is expressed as medians (upper and lower quartiles), all other variables are expressed as mean ± standard deviation or counts (percentages).

### Association between baseline HbA1c levels and the timing of hypertension onset

**Figure 3:**
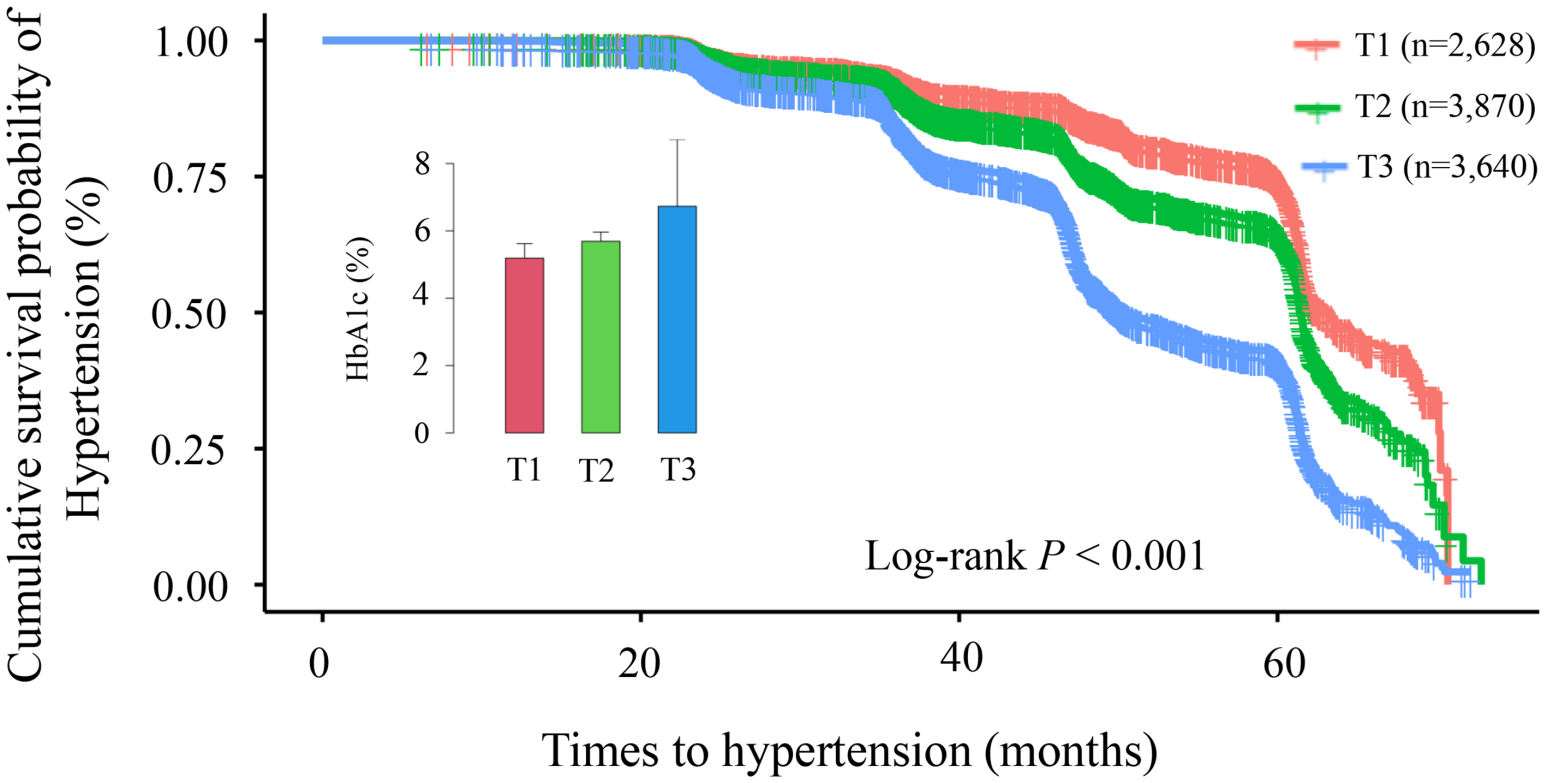
Kaplan–Meier survival curve analysis for hypertension incidence across different tertile groups (T1–T3). The Kaplan–Meier curves demonstrate significant differences in cumulative hypertension incidence among the three groups (log-rank *p* < 0.001). Cumulative incidence rates were: T1 group (red line, *n* = 2,628): 21.84%; T2 group (green line, *n* = 3,870): 28.42%; T3 group (blue line, *n* = 3,640): 48.85%. Over the follow-up period (0–70 months), the survival curves progressively diverged, indicating persistent differences between groups over time. The inset displays a comparison of glycated hemoglobin (HbA1c) levels across groups.

**Figure 4:**
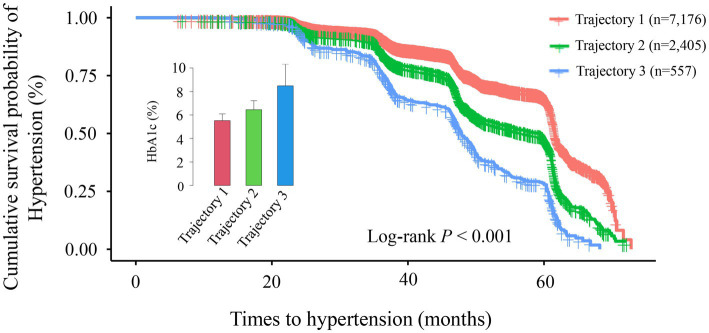
Kaplan–Meier survival curve analysis for hypertension incidence across different trajectory groups (trajectory 1–3). The Kaplan–Meier curves demonstrate significant differences in cumulative hypertension incidence among the three trajectory groups (log-rank *p* < 0.001). Cumulative incidence rates were: Trajectory 1 (red line, *n* = 7,176): 26.77%; Trajectory 2 (green line, *n* = 2,405): 48.36%; Trajectory 3 (blue line, *n* = 557): 66.07%. Over the follow-up period (0–70 months), the survival curves progressively diverged, indicating persistent differences between groups over time. The inset displays a comparison of glycated hemoglobin (HbA1c) levels across trajectory groups.

This study included 40,905 health examination records from 10,138 participants, with an average follow-up duration of 43.92 months (median: 46.28 months; range: 14.17–72.80 months). During this period, 3,452 participants developed hypertension. To assess how different classification methods influence hypertension risk prediction, we employed two distinct approaches: baseline HbA1c tertile analysis (static classification) and trajectory analysis based on longitudinal HbA1c changes (dynamic classification). Kaplan–Meier survival curves for these two classification methods are presented in Figures 3, 4. In the baseline tertile analysis (Figure 3), participants were evenly divided into three groups: T1 (*n* = 2,628), T2 (*n* = 3,870), and T3 (*n* = 3,640). There were significant differences in cumulative hypertension incidence among these groups (log-rank test, *p* < 0.001). The highest HbA1c tertile group (T3) had the highest cumulative incidence of hypertension (48.85%), compared with the intermediate group (T2, 28.42%) and the lowest group (T1, 21.84%). Mean HbA1c levels for each tertile were 5.19% (T1), 5.69% (T2), and 6.73% (T3), respectively. In contrast, trajectory analysis (Figure 4), which classified participants based on longitudinal HbA1c patterns, identified three distinct groups with different population distributions: Trajectory 1 (*n* = 7,176), Trajectory 2 (*n* = 2,405), and trajectory 3 (*n* = 557). Although Trajectory 3 comprised only a small subset of participants (5.49% of the total population), this group exhibited a markedly elevated hypertension risk, with a cumulative incidence of 66.07%. This incidence was substantially higher than that observed in Trajectory 2 (48.36%) and trajectory 1 (26.77%) (log-rank test, *p* < 0.001). Notably, the average HbA1c level in trajectory 3 was 8.42%, significantly higher than any group identified through baseline tertile analysis.

**Figure 5:**
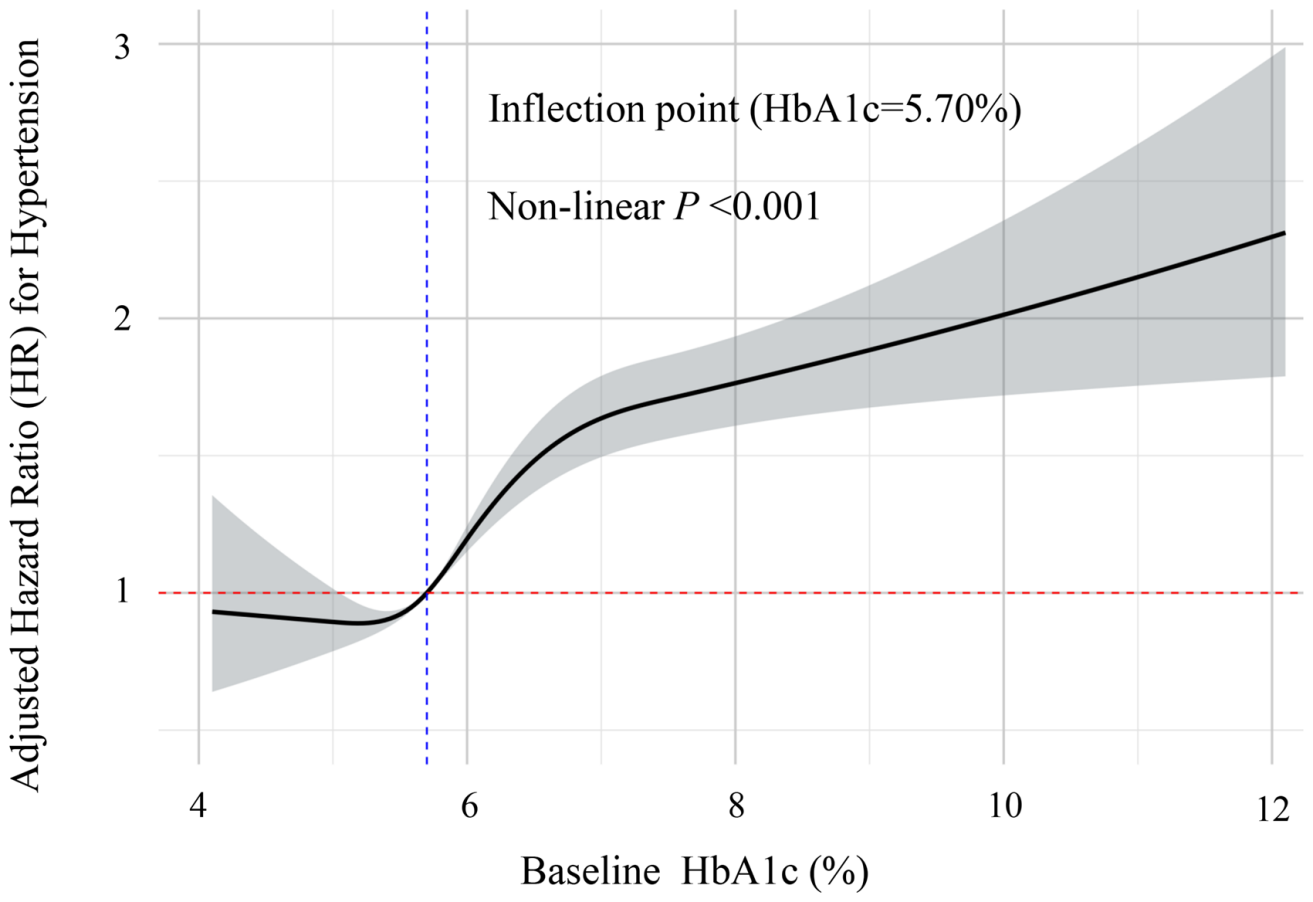
Restricted cubic spline analysis of the non-linear dose–response relationship between baseline HbA1c levels and risk of hypertension. This figure demonstrates the non-linear association between HbA1c levels and the risk of incident hypertension after full adjustment for confounders. The solid line represents the adjusted hazard ratio (HR) estimates, with the gray-shaded area indicating the 95% confidence interval. The analysis identified a significant non-linear inflection point at HbA1c = 5.70% (marked by the blue vertical dashed line). Below this threshold, the relationship with hypertension risk remained relatively stable, while above 5.70%, the risk of hypertension increased significantly with rising HbA1c levels (non-linear *p* < 0.001). The red horizontal dashed line represents the reference risk level (HR = 1.0).

Cox proportional hazards models demonstrated a significant positive association between baseline HbA1c levels and hypertension risk. Compared with participants in the lowest HbA1c tertile (T1), those in the highest tertile (T3) exhibited substantially increased risk (HR = 3.42, 95% CI: 3.05–3.83). This association remained significant even after full adjustment for potential confounders. In the fully adjusted model, the hazard ratio (HR) for T3 versus T1 was 1.49 (95% CI: 1.31–1.70), whereas no significant difference was observed for the T2 group compared with T1. As summarized in Table 3, individuals in the highest tertile (T3) had a 49% greater risk of hypertension compared with the lowest tertile (T1). Sensitivity analyses confirmed these findings. Specifically, when mean HbA1c was included as an additional covariate in the fully adjusted model, the association remained consistent (Supplementary Table S2). Furthermore, after excluding participants who were taking antihyperlipidemic or lipid-lowering medications, the Cox proportional hazards analysis continued to show consistent results (Supplementary Table S3). Additionally, sex-stratified analyses revealed consistent associations between HbA1c trajectories and hypertension risk in both males and females, with no significant interaction observed between sex and HbA1c trajectory (*P* for interaction = 0.5687, Supplementary Table S4), indicating that our findings are robust across different sex groups despite the relatively lower proportion of female participants. Restricted cubic spline analysis illustrated a dose–response relationship between baseline HbA1c and hypertension risk in this health-screening population (Figure 5). Results indicated a significant nonlinear relationship (*P* for nonlinearity < 0.001), with a clear statistical inflection point observed at an HbA1c level of 5.70%. Above this risk prediction threshold (HbA1c ≥ 5.70%), the risk of developing hypertension markedly increased.

**Table 3:** Cox proportional hazards regression analysis for incident hypertension.

HbA1c tertiles / trajectory	Non-adjusted	Model I	Model II
	HR (95% CI) *P-*value	HR (95% CI) *P-*value	HR (95% CI) *P-*value
HbA1c tertiles			
T1	Reference	Reference	Reference
T2	1.42 (1.27, 1.60) < 0.001	1.08 (0.96, 1.23) 0.205	0.99 (0.87, 1.13) 0.897
T3	3.42 (3.05, 3.83) < 0.001	1.85 (1.64, 2.09) < 0.001	1.49 (1.31, 1.70) < 0.001
HbA1c trajectory			
Trajectory 1	Reference	Reference	Reference
Trajectory 2	2.56 (2.33, 2.82) < 0.001	1.60 (1.44, 1.78) < 0.001	1.38 (1.24, 1.53) < 0.001
Trajectory 3	5.33 (4.44, 6.40) < 0.001	3.07 (2.53, 3.73) < 0.001	2.71 (2.21, 3.32) < 0.001

Non-adjusted model adjust for: None.

Model I adjust for: sex, age, ethnic group, and marriage status.

Model II adjust for: sex, age, ethnic group, marriage status, current drinking, current smoking, antihyperlipidemic agents, lipid-lowering medications, BMI, BUN, and eGFR, lymphocyte, neutrophil, LDL-C, TG, and HDL-C. HR, Hazard Ratio; 95%CI, 95% Confidence Interval.

### Relationship between changes in HbA1c trajectory and the occurrence of hypertension events

Using trajectory 1 as the reference, we constructed three models to examine the relationship between HbA1c trajectories and hypertension incidence (Table 3). In the unadjusted model, a positive association was observed between HbA1c trajectory and hypertension risk, with significantly increased risk in trajectory 2 (HR = 2.56, 95% CI: 2.33–2.82) and trajectory 3 (HR = 5.33, 95% CI: 4.44–6.40). After adjusting for key demographic variables (Model I), the positive association persisted, with the hypertension risk in trajectory 2 and 3 increasing by 60% (HR = 1.60) and 207% (HR = 3.07), respectively. Upon further adjustment for all identified confounders (Model II), the relationship remained robust, showing that trajectory 2 (HR = 1.38, 95% CI: 1.24–1.53) and Trajectory Group 3 (HR = 2.71, 95% CI: 2.21–3.32) retained significantly elevated hypertension risks compared with trajectory 1. Sensitivity analyses further confirmed the robustness of these findings, consistently showing a positive association between trajectory 2 and 3 and hypertension risk (Supplementary Table S5). Additionally, comparisons of predictive performance, including incidence rates and hazard ratios—between baseline HbA1c tertiles and trajectory-based classifications, are provided in Supplementary Table S6. To address potential time bias in hypertension onset detection, we conducted logistic regression analyses using hypertension occurrence as a binary outcome. The results demonstrated good consistency with our Cox regression findings (Supplementary Table S7). These findings confirm that our main conclusions remain robust regardless of the statistical approach used and are not substantially affected by potential time bias in hypertension onset detection.

**Figure 6:**
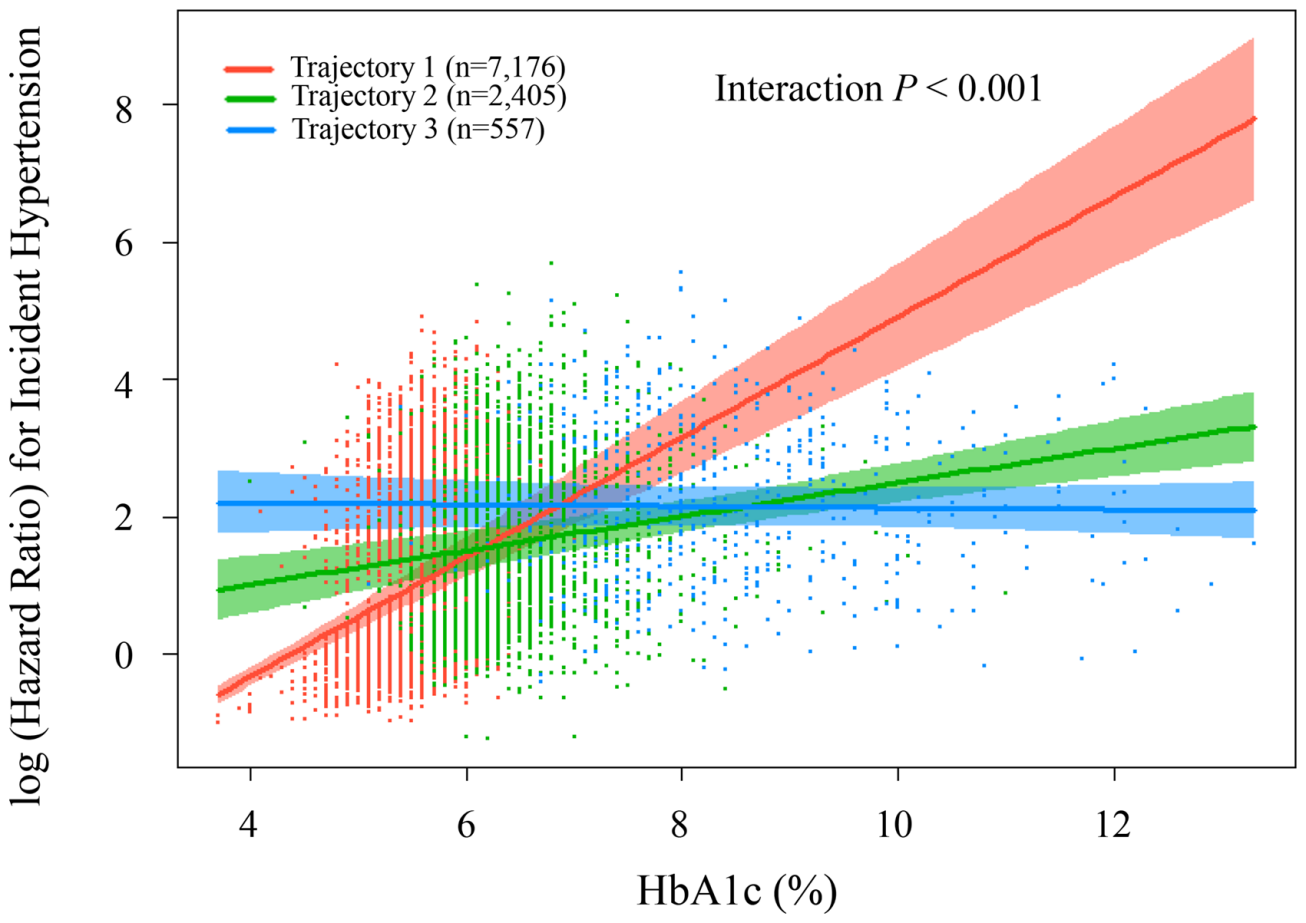
Association between HbA1c levels and risk of incident hypertension by trajectorys. The plot shows log-transformed hazard ratios (with 95% confidence intervals) for incident hypertension according to HbA1c levels, stratified by baseline HbA1c trajectory groups: low-stable (trajectory 1, red, *n* = 7,176, mean HbA1c = 5.57 ± 0.36%), medium-stable (trajectory 2, green, *n* = 2,405, mean HbA1c = 6.45 ± 0.59%), and high-stable (trajectory 3, blue, *n* = 557, mean HbA1c = 8.42 ± 1.39%). Estimates were derived using Cox restricted cubic spline model, adjusted for sex, age, ethnic group, marriage status, current drinking, current smoking, antihyperlipidemic agents, lipid-lowering medications, BMI, BUN, and eGFR, lymphocyte, neutrophil, LDL-C, TG, and HDL-C. Scatter points represent individual observations.

Subsequently, we utilized a Cox regression model with restricted cubic splines to further examine the relationship between HbA1c trajectories and hypertension risk. The analysis identified a significant interaction among the three distinct trajectory groups (*p* < 0.001). Trajectory 1 (red line, *n* = 7,176) demonstrated a marked increase in hypertension risk with rising HbA1c levels, particularly accelerating after HbA1c surpassed 6%. When HbA1c reached approximately 12–13%, the logarithmic hazard ratio approached 8, indicating a substantial escalation in hypertension risk. Participants within this trajectory may represent individuals highly sensitive to elevated blood glucose levels. Trajectory 2 (green line, *n* = 2,405) displayed a moderate positive relationship between HbA1c and hypertension risk. The association was relatively gradual; as HbA1c levels increased from 4 to 12%, the logarithmic hazard ratio rose moderately from approximately 1 to around 3.5. Trajectory 3 (blue line, *n* = 557) showed a relatively stable pattern, maintaining consistently high hypertension risk (log hazard ratio around 2.0) across various HbA1c levels. This indicates that hypertension risk in this group was minimally influenced by fluctuations in HbA1c levels. These findings suggest that the three trajectory groups represent distinct patterns of association between blood glucose and blood pressure within the study population. Particularly for individuals in trajectory 1, there was a pronounced link between HbA1c and hypertension risk, indicating this subgroup could derive substantial benefits from improved glucose management for hypertension prevention. These relationships are clearly illustrated in Figure 6.

## Discussion

The main finding of this study is that both baseline HbA1c levels and longitudinal HbA1c trajectories are significantly associated with the risk of hypertension. In this large-scale retrospective cohort study, we observed a clear gradient relationship between rising baseline HbA1c levels and increased hypertension risk. More importantly, using latent class trajectory modeling, we extend previous cross-sectional and single time-point analyses by demonstrating that distinct HbA1c trajectory patterns, particularly moderate-stable and high-stable trajectories, serve as independent predictors for hypertension development in a health screening population, even after adjusting for traditional risk factors. Notably, we identified a significant difference among three trajectory groups regarding the relationship between HbA1c and hypertension risk (interaction *p* < 0.001). Participants in the low-stable trajectory group (trajectory 1) showed the strongest association between blood glucose and hypertension, suggesting they may benefit substantially from aggressive glucose management strategies. Building upon established findings from major prospective studies—including the ARIC Study, which demonstrated a 14% increased hypertension risk (HR 1.14, 95% CI 1.06–1.23) in the prediabetic HbA1c range (5.7–6.4%) compared to <5.7% (20), and NHANES data (2011–2018) showing elevated hypertension risk around the prediabetic threshold (15). Our large-scale longitudinal study contributes to this evidence base by confirming the 5.70% threshold in a Chinese health screening population and uniquely combining baseline HbA1c analysis with trajectory modeling. These findings extend current knowledge about glucose metabolism and hypertension by providing insights into both static and dynamic glycemic patterns for stratified clinical intervention strategies.

Previous research has consistently demonstrated a strong association between HbA1c levels and hypertension risk (11, 21–23), and our findings further emphasize HbA1c’s predictive value for hypertension incidence. Specifically, we found that higher HbA1c levels were associated with a higher incidence of hypertension. The high-stable HbA1c trajectory group exhibited the highest hypertension incidence (66.07%), notably higher than the highest baseline HbA1c tertile group (T3, 48.85%). Furthermore, the mean HbA1c level in the high-stable group was significantly higher (8.42%) compared to the T3 group (6.60%), suggesting that markedly elevated HbA1c may contribute directly to hypertension onset. Our results align well with previous studies. For instance, a two-year observational study involving 4,074 participants from the China Health and Nutrition Survey database reported a significant linear relationship between HbA1c levels and hypertension incidence after adjusting for confounders (14). Similarly, a study involving 10,503 participants from the U. S. NHANES (2011–2018) found an independent effect of HbA1c on hypertension risk, with prevalence increasing significantly alongside HbA1c levels (15). However, our findings differ from a large longitudinal study conducted in Japan, which found no independent association between HbA1c and incident hypertension (24). This discrepancy may be attributed to differences in study populations, as the Japanese study excluded individuals with known diabetes. Methodological variations in HbA1c measurement may also have contributed to differences in outcomes, affecting comparability between studies.

Furthermore, our study identified a clear, positive, and nonlinear association between HbA1c levels and hypertension risk. Specifically, hypertension risk significantly increased after reaching a threshold HbA1c of 5.70%. This finding closely aligns with the results from a dose–response meta-analysis by Zhong et al. (25), who also observed a relatively flat cardiovascular risk curve at HbA1c levels below 5.70%, followed by a steep increase beyond this threshold. The critical inflection points at 5.70% identified through our restricted cubic spline analysis closely matches the value reported by Zhong et al. (25) reinforcing its validity and clinical relevance across diverse populations. Notably, clinical practice currently defines HbA1c values below 5.70% as normal, values between 5.70 and 6.40% as prediabetic, and values ≥6.50% as indicative of diabetes (26). Our results suggest cardiovascular damage could begin at HbA1c levels below the conventional diagnostic threshold (6.50%), highlighting important implications for preventive hypertension strategies.

Using LCTM, our study identified three distinct HbA1c trajectory patterns, each closely associated with hypertension risk. Notably, the high-stable trajectory group (trajectory 3) demonstrated the highest risk for developing hypertension (HR = 2.71, 95% CI: 2.21–3.32), suggesting that persistently elevated HbA1c is an important predictor of hypertension. This finding aligns with previous research by Liu et al. (16), who also reported significant associations between baseline HbA1c and its longitudinal changes with hypertension risk in a three-year cohort study of 6,546 participants. Similarly, the Atherosclerosis Risk in Communities study by Bower et al. (20) reported 21 and 51% increased hypertension risks in participants with HbA1c levels of 5.70–6.40% and ≥6.50%, respectively, compared to those below 5.70%, further supporting our findings. Our study builds on this prior evidence through a larger sample size and a longer average follow-up period (43.92 months), enhancing statistical reliability and generalizability. Importantly, our comprehensive analysis simultaneously considered baseline HbA1c and HbA1c trajectories, yielding higher hazard ratios in trajectory analysis (trajectory 3 vs. trajectory 1: HR = 2.71) than static baseline tertile comparisons (T3 vs. T1: HR = 1.49). This indicates trajectory analysis provides superior predictive insight compared to single baseline measurements, consistent with findings by Wang et al. (27). The observed dose–response relationship between HbA1c trajectories and hypertension risk likely reflects multiple pathophysiological mechanisms converging to elevate blood pressure. The relationship between sustained hyperglycemia and hypertension risk in our high-stable trajectory group represents a complex interplay of pathological and therapeutic factors. While sustained hyperglycemia induces endothelial dysfunction through reduced nitric oxide bioavailability, consistent with Chen et al. (28, 29) findings that endothelial dysfunction plays a key role in increasing cardiovascular risk in type 2 diabetes, participants with HbA1c levels of 6–13% would inevitably receive antidiabetic medications with modest blood pressure-lowering effects (SGLT2 inhibitors, GLP-1 agonists, DPP-4 inhibitors). Our observed increased hypertension risk in the high-stable group suggests that the adverse metabolic effects of sustained hyperglycemia exceed the cardiovascular protective benefits of standard pharmacological interventions. This process occurs concurrently with increased oxidative stress and inflammatory responses, which compromise vascular integrity through formation of AGEs and heightened reactive oxygen species production. Lamprea-Montealegre and Goh demonstrated that AGEs contribute significantly to arterial stiffness through intermolecular collagen cross-linking (30, 31), explaining our observation of substantially higher hypertension risk in the high-stable trajectory group. Chronic glycemic elevation simultaneously activates the RAAS and sympathetic nervous system, as Sinha and Haque (32) highlighted in their comprehensive review. Przezak et al. (33) provided direct evidence that glycated hemoglobin correlates with arterial stiffness and endothelial dysfunction in patients with resistant hypertension and uncontrolled diabetes, supporting our findings of HbA1c-related hypertension risk. Additionally, Peker and Boyraz (34) demonstrated that plasma AGEs interact with sodium dietary intake and renal handling, potentially explaining the synergistic effect observed in our high-stable trajectory group, where the hypertension risk (HR = 2·71, 95% CI: 2·21–3·32) substantially exceeded that of the medium-stable trajectory group (HR = 1·38, 95% CI: 1·24–1·53). These findings underscore the value of monitoring longitudinal glycemic trajectories rather than single-point measurements for hypertension risk stratification, particularly at HbA1c thresholds above 5·70%.

Additionally, the three distinct HbA1c trajectories we identified highlight diverse glucose–blood pressure relationships. Particularly, trajectory 1 showed the strongest association, suggesting that individuals in this subgroup could significantly benefit from enhanced glucose control strategies for hypertension prevention, echoing similar observations by Yang et al. (35), who reported pronounced blood pressure improvements from glycemic control within specific subpopulations. By applying LCTM to categorize participants into distinct HbA1c trajectory groups, this study captures longitudinal changes more effectively than conventional classification methods. This innovative approach provides a valuable framework for individualized clinical risk assessment based on dynamic metabolic changes. However, our findings differ from those reported by Britton et al. (36) in their large prospective cohort study of 19,858 women, which found that the association between HbA1c and hypertension risk became non-significant after adjusting for BMI. This discrepancy might be attributed to several factors: firstly, differences in study populations, as Britton et al. (36) only included women, while men comprised 68.56% of our cohort; secondly, significant differences in follow-up duration (11.6 years vs. 3.7 years); and thirdly, variations in the confounding factors adjusted for. Our study comprehensively controlled for metabolic factors including BUN, creatinine, eGFR, fasting blood glucose, lymphocytes, neutrophils, WBC, LDL cholesterol, triglycerides, and HDL cholesterol, which may have provided more thorough control of potential confounding effects.

The clinical significance of our study lies in providing novel insights for accurately identifying individuals at high risk of developing hypertension. Firstly, the identification of an HbA1c inflection point at 5.70% represents an epidemiological risk marker for hypertension development rather than a diagnostic cutoff, offering clinicians a risk stratification tool for screening purposes. This statistical threshold, derived from spline regression analysis, differs in nature from established diagnostic categories for diabetes and prediabetes (normal <5.7%, prediabetes 5.7–6.4%, diabetes ≥6.5%) (25). The finding highlights that even within the upper-normal glycemic range, HbA1c may serve as an early risk predictor of hypertension for population health surveillance. Secondly, the identification of three distinct HbA1c trajectory patterns, particularly trajectory 1, representing individuals highly sensitive to increasing glucose levels, provides theoretical support for clinical risk stratification. Compared with conventional single-point measurements, trajectory analysis based on long-term HbA1c dynamics may more precisely reflect individual metabolic profiles and associated risk (37). Based on our findings, from a risk stratification perspective, enhanced blood pressure surveillance may be considered among individuals with HbA1c levels above 5.70%, complementing current approaches that focus primarily on diabetic patients. Those with HbA1c values in the high-normal range (5.70–6.40%) showing upward trends over multiple assessments may particularly benefit from more frequent monitoring and proactive lifestyle interventions. From a public health perspective, incorporating HbA1c measurements into routine health screenings could facilitate early identification of cardiometabolic risks (38), especially among individuals with a family history of hypertension or other relevant risk factors. Furthermore, an important finding from our trajectory analysis is that the three HbA1c trajectory groups maintained largely parallel patterns throughout the follow-up period, with minimal crossing between trajectories. This parallel nature provides novel evidence supporting the cost-effectiveness of baseline HbA1c measurement for hypertension risk assessment. Since individuals tend to maintain relatively stable HbA1c levels within their respective trajectory groups over time, baseline measurements may provide sufficient risk stratification information for most clinical purposes, potentially reducing the need for repeated HbA1c assessments in routine screening. This finding has important implications for healthcare resource allocation and supports more streamlined, cost-effective screening strategies, particularly in resource-limited settings. However, the implementation of these recommendations should consider population-specific factors. In populations with limited healthcare access or lower socioeconomic status, the HbA1c thresholds and intervention strategies may need adjustment based on available resources and population-specific risk profiles. Healthcare providers should be aware that the strength of HbA1c-hypertension associations and the effectiveness of preventive interventions may vary across different socioeconomic contexts. When interpreting our findings clinically, it is important to recognize that patients with elevated HbA1c typically receive antidiabetic medications with cardiovascular benefits. The observed HbA1c-hypertension associations reflect the net effect of both metabolic dysfunction and therapeutic interventions, suggesting that sustained hyperglycemia remains a significant cardiovascular risk factor despite standard pharmacological management. Future research should explore the stability and predictive performance of trajectory-based classification in diverse populations, accounting for variations in age, sex, and ethnicity. Additionally, cost-effectiveness and long-term efficacy of personalized interventions tailored to specific HbA1c trajectories warrant further evaluation. Investigating the causal relationship and underlying mechanisms between HbA1c fluctuations and hypertension development will also be critical for developing targeted preventive and therapeutic strategies. What’s more, investigating the combined predictive accuracy of HbA1c trajectories with inflammatory markers such as neutrophil-lymphocyte ratio may further enhance risk stratification capabilities.

### Strengths and limitations

The primary strengths of this study include the availability of extensive longitudinal follow-up data, which provides robust statistical power. To our knowledge, this is the first study to quantitatively examine both baseline HbA1c levels and their longitudinal trajectories in relation to hypertension incidence within a health screening population, highlighting the potential of HbA1c as a predictive tool for hypertension risk. Additionally, repeated HbA1c measurements and follow-up of type 2 diabetes outcomes allowed exploration of potential causal relationships. However, several limitations must be acknowledged. First, despite the longitudinal design establishing a temporal sequence, the observational nature of the study precludes definitive causal inference. Second, although we adjusted for a wide range of confounders, residual confounding from unmeasured variables remains possible. In particular, our dataset lacked detailed information on dietary patterns, physical activity levels, genetic predisposition, and importantly, changes in lifestyle behaviors during the 7-year follow-up period, which are important determinants of both HbA1c levels and hypertension risk. The absence of data on lifestyle modifications during follow-up (such as dietary interventions, exercise programs, smoking cessation, or stress management) limits our ability to distinguish between HbA1c trajectory changes driven by natural metabolic progression versus those resulting from behavioral interventions. The absence of these baseline lifestyle factors and their dynamic changes may have resulted in residual confounding, potentially affecting the magnitude and precision of the observed HbA1c-hypertension associations. Third, this study was conducted at a single health screening center in China, which may have introduced potential selection bias due to the “healthy user effect.” While the cardiovascular risk profiles of our participants were broadly comparable to those reported in national surveys, individuals who undergo routine health screenings may be more health-conscious and have better access to healthcare services. This may limit the generalizability of our findings to populations with different socioeconomic backgrounds or healthcare access. Specifically, socioeconomic status and healthcare accessibility may modify the HbA1c-hypertension association. Higher socioeconomic status is typically associated with better lifestyle factors and timely medical interventions, which may attenuate the adverse effects of elevated HbA1c. Conversely, in resource-limited settings, abnormal HbA1c levels may demonstrate stronger associations with hypertension risk due to delayed detection and intervention. Future studies should investigate these associations across diverse populations to determine the consistency and modifiability of our findings. Fourth, during the approximately 7-year follow-up period, some participants may not yet have developed hypertension. Moreover, while LCTM provided valuable insights into HbA1c patterns, we acknowledge that the predefined trajectory categories may not fully capture the variability in individual glycemic patterns, and some uncertainty in group classification may exist. Lastly, we did not systematically exclude participants with anemia or hemoglobinopathies that could affect HbA1c measurement accuracy. While our HPLC method can detect common hemoglobin variants and routine screening would identify severe anemia, mild anemia or less common hemoglobin disorders may have introduced measurement variability, though this would likely represent non-differential misclassification across exposure groups. Future studies employing alternative modeling approaches and incorporating data from multiple screening centers with varying healthcare access and extended follow-up durations may yield additional insights into the complex dynamics of glycemic variability and further validate our findings.

## Conclusion

In this longitudinal cohort study based on a health screening population, we confirmed that both baseline HbA1c levels and their dynamic trajectories—particularly moderate-stable and high-stable patterns—are independent risk factors for hypertension development. Notably, we demonstrated a clear association between improved glycemic control and reduced hypertension risk in the low-stable HbA1c trajectory group, extending previous findings through longitudinal trajectory analysis methodology. These findings provide empirical support for risk stratification and early intervention within primary prevention systems. However, the external validity of our results requires further verification in more diverse populations, particularly those with varying healthcare infrastructure and socioeconomic contexts. Future research should specifically examine whether socioeconomic factors and healthcare accessibility modify the strength and clinical significance of HbA1c-hypertension associations to inform broader, more generalizable prevention strategies.

## Data Availability

The datasets presented in this article are not readily available because due to the sensitive nature of health information contained in this dataset, data access is restricted to protect participant privacy and comply with institutional data protection policies. The dataset may be made available upon reasonable request to the corresponding author, subject to approval by the Henan Provincial People’s Hospital Ethics Committee and execution of an appropriate data use agreement. Any data sharing will be conducted in accordance with applicable privacy regulations and institutional guidelines to ensure participant confidentiality is maintained. Requests to access the datasets should be directed to ybsun8788@163.com.
