# Supplementary material for: The impact of glycated hemoglobin trajectories on hypertension risk: a retrospective cohort study
Source: Front Nutr. 2025 Nov 5;12:1680891. doi: 10.3389/fnut.2025.1680891 (PMC12626783; doi:10.3389/fnut.2025.1680891)
Supplement: Supplementary file 1 [file Table_1.docx]

**Table S1.** Baseline characteristics and hypertension incidence in total participants

| **Characteristics** | Total |
| --- | --- |
| **N** | 10,138 |
| **HbA1c** | 5.93 ± 0.88 |
| **Mean AIP** | 5.91 ± 0.83 |
| **Age, years** | 54.03 ± 12.97 |
| **Sex, n (%)** |  |
| Female | 3,187 (31.44) |
| Male | 6,951 (68.56) |
| **Ethnic group, n (%)** |  |
| Non-han | 124 (1.22) |
| Han | 10,014 (98.78) |
| **Marriage status, n (%)** |  |
| Unmarried | 154 (1.52) |
| Married | 9,984 (98.48) |
| **Current drinking, n (%)** |  |
| No | 7,965 (78.57) |
| Yes | 2,173 (21.43) |
| **Current smoking, n (%)** |  |
| No | 8,472 (83.57) |
| Yes | 1,666 (16.43) |
| **Antihyperlipidemic agents, n (%)** |  |
| No | 9,985 (98.49) |
| Yes | 153 (1.51) |
| **Lipid-lowering medications, n (%)** |  |
| No | 9,920 (97.85) |
| Yes | 218 (2.15) |
| **BMI, kg/m^2^** | 24.65 ± 3.00 |
| **SBP, mmHg** | 124.82 ± 16.57 |
| **DBP, mmHg** | 74.25 ± 10.46 |
| **BUN, mmol/L** | 5.23 ± 1.39 |
| **Cre, μmol/L** | 66.42 ± 19.00 |
| **eGFR, mL/min/1.73m^2^** | 110.68 ± 21.58 |
| **FBG, mmol/L** | 5.19 ± 1.29 |
| **Lymphocyte,** **10^9^/L** | 1.91 ± 1.01 |
| **Neutrophil,** **10^9^/L** | 3.29 ± 1.05 |
| **WBC,** **10^9^/L** | 5.73 ± 1.68 |
| **TC, mmol/L** | 4.84 ± 0.96 |
| **LDL-C, mmol/L** | 2.83 ± 0.78 |
| **TG, mmol/L** | 1.45 (1.06-2.05) |
| **HDL-C, mmol/L** | 1.31 ± 0.30 |
| **Time, month** | 43.92 ± 13.76 |
| **Hypertension, n (%)** |  |
| No | 6,686 (65.95) |
| Yes | 3,452 (34.05) |

HbA1c, Glycosylated hemoglobin; BMI, body mass index; SBP, systolic blood pressure; DBP, diastolic blood pressure; BUN, blood urea nitrogen; Cre, Creatinine; eGFR, estimated glomerular filtration rate; FBG, fasting blood glucose; WBC, white blood cell; TC, total cholesterol; LDL-C, low-density lipoprotein cholesterol; TG, triglycerides; HDL-C, high-density lipoprotein cholesterol. Except for TG is expressed as medians (upper and lower quartiles), all other variables are expressed as mean ± standard deviation or counts (percentages).
