# Supplementary material for: The impact of glycated hemoglobin trajectories on hypertension risk: a retrospective cohort study
Source: Front Nutr. 2025 Nov 5;12:1680891. doi: 10.3389/fnut.2025.1680891 (PMC12626783; doi:10.3389/fnut.2025.1680891)
Supplement: Supplementary file 4 [file Table_4.docx]

**Table S4.** Sex-stratified analysis of the association between HbA1c trajectory patterns and incident hypertension

| **HbA1c Trajectory** | **Female** | **Events** |  | **Male** | **Events** |  | ***P* for interaction** |
| --- | --- | --- | --- | --- | --- | --- | --- |
|  | N (%) | (%) | HR (95%CI) | N (%) | (%) | HR (95%CI) |  |
| Trajectory1 | 2,440 (76.56) | 20.00 | Reference | 4,736 (68.13) | 30.26 | Reference | 0.569 |
| Trajectory2 | 651 (20.43) | 47.16 | 1.50 (1.20, 1.87) ^***^ | 1,754 (25.23) | 48.80 | 1.32 (1.17, 1.50) ^***^ |  |
| Trajectory3 | 96 (3.01) | 68.75 | 2.98 (1.77, 5.03) ^***^ | 461 (6.63) | 65.51 | 2.61 (2.09, 3.25) ^***^ |  |

Model adjust for: age, ethnic group, marriage status, current drinking, current smoking, antihyperlipidemic agents, lipid-lowering medications, BMI, BUN, and eGFR, lymphocyte, neutrophil, LDL-C, TG, HDL-C and mean HbA1c. HR, Hazard Ratio; 95%CI, 95% Confidence Interval. ****P* < 0.001
