# Supplementary material for: The impact of glycated hemoglobin trajectories on hypertension risk: a retrospective cohort study
Source: Front Nutr. 2025 Nov 5;12:1680891. doi: 10.3389/fnut.2025.1680891 (PMC12626783; doi:10.3389/fnut.2025.1680891)
Supplement: Supplementary file 5 [file Table_5.docx]

**Table S5.** Sensitivity analysis of the association between trajectories of the HbA1c and hypertension

|  | HR | 95%CI | *P* |
| --- | --- | --- | --- |
| **HbA1c Trajectory** |  |  |  |
| Trajectory1 | Reference |  |  |
| Trajectory2 | 1.28 | 1.10, 1.49 | 0.001 |
| Trajectory3 | 2.12 | 1.42, 3.18 | <0.001 |

Model adjust for: sex, age, ethnic group, marriage status, current drinking, current smoking, antihyperlipidemic agents, lipid-lowering medications, BMI, BUN, and eGFR, lymphocyte, neutrophil, LDL-C, TG, HDL-C and mean HbA1c. HR, Hazard Ratio; 95%CI, 95% Confidence Interval.
