# Supplementary material for: The impact of glycated hemoglobin trajectories on hypertension risk: a retrospective cohort study
Source: Front Nutr. 2025 Nov 5;12:1680891. doi: 10.3389/fnut.2025.1680891 (PMC12626783; doi:10.3389/fnut.2025.1680891)
Supplement: Supplementary file 6 [file Table_6.docx]

**Table S6** Comparison of hypertension incidence rates between baseline HbA1c tertiles and trajectorys

| **Classification Method** | **Groups** | **N (%)** | **Hypertension,n (%)** | **HR (95% CI)** | ***P*** |
| --- | --- | --- | --- | --- | --- |
| **HbA1c Tertiles** | T1 | 2,628 | 574 (21.84) | Reference |  |
|  | T2 | 3,870 | 1,100 (28.42) | 0.99 (0.87, 1.13) | 0.897 |
|  | T3 | 3,640 | 1,778 (48.85) | 1.49 (1.31, 1.70) | <0.001 |
| **Trajectory Groups** | Trajectory 1 | 7,176 | 1,921 (26.77) | Reference |  |
|  | Trajectory 2 | 2,405 | 1,163 (48.36) | 1.38 (1.24, 1.53) | <0.001 |
|  | Trajectory 3 | 557 | 368 (66.07) | 2.71 (2.21, 3.32) | <0.001 |

Adjust for: sex, age, ethnic group, marriage status, current drinking, current smoking, antihyperlipidemic agents, lipid-lowering medications, BMI, BUN, and eGFR, lymphocyte, neutrophil, LDL-C, TG, and HDL-C. HR, Hazard Ratio; 95%CI, 95% Confidence Interval.
