# Supplementary material for: The impact of glycated hemoglobin trajectories on hypertension risk: a retrospective cohort study
Source: Front Nutr. 2025 Nov 5;12:1680891. doi: 10.3389/fnut.2025.1680891 (PMC12626783; doi:10.3389/fnut.2025.1680891)
Supplement: Supplementary file 7 [file Table_7.docx]

**Table S7. Sensitivity analysis using logistic regression for incident hypertension**

| **Variables** | **Non-adjusted**  **OR (95% CI)** | ***P*-value** | **Model I**  **OR (95% CI)** | ***P*-value** | **Model II**  **OR (95% CI)** | ***P*-value** |
| --- | --- | --- | --- | --- | --- | --- |
| **HbA1c tertiles** |  |  |  |  |  |  |
| T1 | Reference |  | Reference |  | Reference |  |
| T2 | 1.44 (1.31, 1.60) | <0.001 | 1.23 (1.11, 1.36) | <0.001 | 1.13 (1.02, 1.25) | 0.018 |
| T3 | 2.76 (2.51, 3.04) | <0.001 | 1.88 (1.71, 2.08) | <0.001 | 1.62 (1.47, 1.79) | <0.001 |
| **HbA1c Trajectories** |  |  |  |  |  |  |
| Trajectory 1 | Reference |  | Reference |  | Reference |  |
| Trajectory 2 | 1.76 (1.64, 1.89) | <0.001 | 1.30 (1.21, 1.40) | <0.001 | 1.17 (1.08, 1.26) | <0.001 |
| Trajectory 3 | 2.98 (2.66, 3.33) | <0.001 | 1.91 (1.70, 2.14) | <0.001 | 1.74 (1.54, 1.96) | <0.001 |

Non-adjusted model adjust for: None.

Model I adjust for: sex, age, ethnic group, and marriage status.

Model II adjust for: sex, age, ethnic group, marriage status, current drinking, current smoking, antihyperlipidemic agents, lipid-lowering medications, BMI, BUN, and eGFR, lymphocyte, neutrophil, LDL-C, TG, and HDL-C. HR, Hazard Ratio; 95%CI, 95% Confidence Interval.
